# Midwife students and hospital mentors satisfaction with professional practice in Estonia

**DOI:** 10.18332/ejm/145790

**Published:** 2022-02-25

**Authors:** Kaire Sildver, Marika Merits, Anna Makaronskaja, Cathlin Pilliroog, Hanna-Maria Aavik, Hanna-Maria Trei

**Affiliations:** 1 Midwifery Department, Health Education Centre, Tallinn Health Care College, Tallinn, Estonia

**Keywords:** midwife students, hospital mentors, satisfaction, professional practice

## Abstract

**INTRODUCTION:**

Mentoring has played an important role in the development and support of healthcare students during the last 15 years. Constant monitoring performed by a mentor and their constructive feedback is a useful tool in professional practice. The greatest factor having a negative influence is lack of time. Mentors and midwifery students’ satisfaction with practical training can be significantly affected by the clinical practice environment. This study aims to analyze the satisfaction of the mentors at women's clinics and midwifery students with the clinical practice.

**METHODS:**

The mixed methods study included midwifery mentors from the women's clinic and students who completed internships at the clinic in Estonia. The duration of the study was three years (2016–2019). Mentors were surveyed through focus group interviews. Students were surveyed through a semi-closed questionnaire. The study involved 15 midwives and 127 midwifery students.

**RESULTS:**

The mentors are aware that their responsibilities include the instruction, training, and assessment of the trainees, and they believe that a safe environment has an important role in passing the training successfully. Additionally, the most challenging aspect of providing instruction from the view of the mentors is the resultant lack of time. Students are satisfied with the mentors, co-workers, wards, and overall practical training at the women’s clinic.

**CONCLUSIONS:**

The most challenging aspect of providing instruction from the view of the mentors is the resultant lack of time. Students’ satisfaction with the said mentors is based on how well the students thought cooperation worked amongst mentors and co-workers. It is a problem for students from time to time that they are expected to have higher levels of skills than the knowledge they have acquired allows.

## INTRODUCTION

Mentors and mentoring are central to the internship environment. A mentor is a smart and reliable counselor and supervisor who stays with the intern at the beginning of their career and supports them during the work process^1^. Over the past years, mentoring has played an important role in the development and support of healthcare students^2^.

In 2005, the European Parliament adopted a professional standard for midwives setting out European Union directives^3^. The professional qualification requirements for midwives have been established by the International Confederation of Midwives^4^. According to the directives of the European Union, a student majoring in midwifery is required to complete at least three years of study, which includes theoretical and practical training. Training as a midwife must include at least 40% theory and 50% practice^5^. In Estonia, professional higher education in midwifery lasts four and a half years with the award of 270 ECTS (European Credit Transfer and Accumulation System). Graduate midwives acquire the profession of a midwife and the degree of Bachelor of Science in Health Sciences (BSc). The degree entitles them to work in various fields of healthcare as both midwives and nurses^6^.

The International Code of Ethics for Midwives states that the transfer of professional knowledge is essential for the advancement of the field^7^. In several countries, a model of internship mentoring is used to support the student’s professional development and assessment process. Mentoring is considered to be an individual educational process that connects a specialist with a person of little experience in order to promote the latter’s professional development^8^.

The organization of the internship and the successful completion of the student's internship is important for the higher education institution, the student, the internship institution and the best possible maternity care. In order to achieve good results, the cooperation of all parties (student, internship base, and school supervisor), a suitable environment, and dedication of time are needed^9^. Based on the student’s professional development, it is important to achieve a working relationship with mentors^10^, and one of the success factors is continuous and thorough feedback throughout the internship^11^. The literature repeatedly emphasizes that lack of time is one of the negative factors of the atmosphere^12^. This study aims to analyze the satisfaction of the mentors at women's clinics and midwifery students with the clinical practice.

## METHODS

This mixed-methods study has been conducted in Tallinn, Estonia, where midwifery mentors from one women’s clinic and midwifery students who completed internships at this clinic were recruited. The duration of the study was three years (2016–2019), in order to collect enough data.

The qualitative method for researching midwives was used because this topic has not been studied in the Estonian context before, and it is important to obtain more information. Focus group interviews are often employed simply because this is a quick and suitable way to collect data from several subjects simultaneously^13,14^. The study involved 15 mentors of the women’s clinic, who were questioned through focus group interviews. Three mentors had 1–5 years of mentoring experience, nine had 5–15 years of experience, and three had 15–35 years of experience. The average duration of interviews for each focus group was 1.5 hours, and a total of three groups were interviewed. The mentoring methods of midwifery mentors and the opinion of mentors regarding the influence of mentors, co-workers, and the atmosphere of the professional practice of midwifery students were analyzed. There were 15 interview questions used in the study, two of which were related to background data, and the remaining questions were divided into the following groups as sub-topics: student, mentor, and co-workers; atmosphere (department); and organization of internship. Qualitative content analysis was used to process the responses to the interviews, while the narrower method was a conversational analysis based on an inductive approach. Interviews were transcribed, grouped, and analyzed inductively and deductively.

The midwifery students were studied quantitatively. A total of 127 second-year and fifth-year students participated in the study, as first-year students have only theory studies. The numerical data were collected by a semi-closed questionnaire, which used a 5-point Likert scale^15^, in addition to which it was possible to comment on one’s answer.

All students who passed an internship at the respective hospital during this time had the opportunity to participate in the study. Professional internships were considered to be internships within the framework of this research, where the main objectives were to develop the student’s professional skills and knowledge in the field of pregnancy, childbirth, the postpartum period, neonatology, or gynecological diseases. The questionnaire was distributed to students on paper and contained 38 statements in which students were asked to rate on a five-point scale whether the statements were valid in case of their hospital internship: ‘5’ in the questionnaire indicated the full validity of the statement; ‘3’ indicated the center of the scale, i.e. ‘can't say’, and ‘1’ indicated the complete invalidity in case of the student’s hospital internship. Students also had the opportunity to write additional/explanatory comments under the statements and make suggestions for improving the quality of internships. The 14 statements were thematically divided into three thematic blocks: 1) Student satisfaction and evaluation of supervision; 2) Satisfaction, and evaluation of the work of the department and co-workers; and 3) Student satisfaction and evaluation of practice.

Ethical approval was obtained from the college and the research center of the women’s clinic to conduct the research. All study participants participated voluntarily, and they were informed about the aims of the study and the need for the study. Participants read the informed consent sheet, signed it, giving their consent to use the data anonymously. Participants also had the opportunity to opt-out at any stage during the study. The data were stored on a password-protected medium and kept in a safe. Confidentiality was guaranteed for all study participants.

## RESULTS

### Supervisors’ satisfaction and assessment of student supervision

Midwifery mentors working at the women’s clinic described that a student has usually one mentor in the delivery room, but when a mentor falls ill or on a day when there are few patients, the intern is also supervised by other co-workers.

Mentors find that mentoring would be more effective if interns set personal goals in addition to learning outcomes and revise what they had learned before entering the work environment. It was also pointed out that interns should find out what skills and knowledge they want to acquire.

The attitude of mentors to the intern is influenced by various factors. The intern’s attitude, activity, proper appearance, and preparation were highlighted. According to several mentors, their attitudes towards the intern are influenced by the first impressions and personal qualities, such as empathy, responsibility, and ethical attitudes. In their opinion, the first impression is influenced by accuracy and correctness when the intern arrives at the internship.

*‘The most important thing for me is that the interns are motivated. When they take the initiative, they know – we'll get along. If they are not interested, I have to ask them to participate, then from that moment on, any relationship between us is over, a person must want it themselves, I am not obliged to teach anyone who does not want to learn.’* (Mentor 14)

Mentors acknowledge that interns adapt and implement mentor’s attitudes and behaviors when working later. Therefore, the mentor can pass on work methods based on personal experience for a patient-centered approach and the best solutions for different situations.

*‘It is still the case that if we are a role model, a teacher, she will understand this midwifery. Well, it's like, she puts together, so to speak, the things she learns in school. It's really thanks to us then.’* (Mentor 1)

Most mentors assume the interns are already motivated and full of enthusiasm when they come to the internship. They motivate the intern primarily with praise and recognition. Mentors also believe that encouragement and support help the intern to develop professionally. Patients’ gratitude and a motivated and committed team are also considered a source of motivation. According to the mentors, the interns are also motivated by trust and greater responsibility.

Mentors expect interns to have a good level of theoretical knowledge and manual skills that correspond to the course. It is assumed that the intern is motivated and complements and develops his/her own skills with each internship. If the basic knowledge is not sufficient, this causes difficulties for the mentor in mentoring. Mentors have noticed the increasing use of smart devices in the internship. According to the mentor, this is a big problem because the use of smartphones reduces the intern's activity, attention, and ability to acquire new knowledge. On the positive side, if there is a lot of work in the department, it is very good to have interns to help.

All mentors of the study preferred to provide feedback to the intern immediately after the incident or action. It is considered that immediate feedback allows one to get to the heart of the situation, and there is no risk that the details of the situation will be forgotten.

Mentors believe that creating a safe environment is essential for a successful internship. The mentors unanimously agree that the internal climate of the department depends not only on the mentor and the intern but also on the patients and the entire hospital staff and management. Mentors find that sound procedures and rules create a stable and positive internal climate in the department and, thus, throughout the hospital.

It is not possible to fully plan the workload of the hospital. Therefore, the time factor emerged as a single negative internal climate factor in all focus groups. Due to lack of time, the mentor may have a number of tasks that need to be solved immediately. Mentors state that additional time is needed to carry out the activities with the intern and to supervise him/her at the same time. This can create a situation where it is easier for the mentor to perform the tasks himself/herself and let the intern monitor them from the side. Mentors lack a fixed amount of time, which allows interns to meet privately with their mentors before the start of the internship and repeatedly during the internship. Mentors think that meetings could be part of the job tasks and that a safe environment, i.e. a private space for the conversation between the mentor and the intern, should be provided.

*‘We actually have a large number of students, in fact, so we have a large workload, and we still have students almost all year round in the house.’* (Mentor 1)

*‘But when I already have several things on my back, I end up doing it myself fast, which doesn't really help [shakes her head] in the process of learning, that she just stands looking on there.’* (Mentor 2)

Mentors are motivated to supervise by an intern who is active, prepared, punctual, interested in self-improvement, and wishes to apply the previously acquired theoretical knowledge in the internship base. This, in turn, creates a desire in the mentor to pass on knowledge, involve the trainee in work, and be an exemplary mentor. Demotivating factors are the abundance of interns, their lack of activity, or their unprofessional attitude. Mentors point out that in such a case, they would rather do their job themselves than ask the intern repeatedly. They unanimously agreed that financial rewards also motivate mentoring, but this does not affect quality, as mentors still do their best without any extra fee. Mentors also find that they would be very interested in seeing study and working conditions abroad, i.e. that professional in-service training and conferences abroad would add motivation. Of the mentors who supervise midwifery internships, not everyone feels that mentoring is suitable for them, and, if possible, they would prefer not to supervise. The reasons given are, for example, the lack of personal qualities required for the role of a mentor or little work experience. Despite personal preferences, their attitude towards mentoring is conscientious, and the role of the mentor is performed willingly. In connection with the supervision of foreign students, some mentors point out that compared to the Estonian interns, the language barrier makes supervision more difficult. This can be due to the level of language skills of both the mentor and the intern. The lack of English language skills of a midwife intern from another country increases the workload of the mentor. But there are mentors who think that being a mentor for international students is an exciting and interesting challenge.

*‘… and with international students, it's also very good, very interesting.’*

### Student satisfaction and assessment of supervision

**Figure 1:**
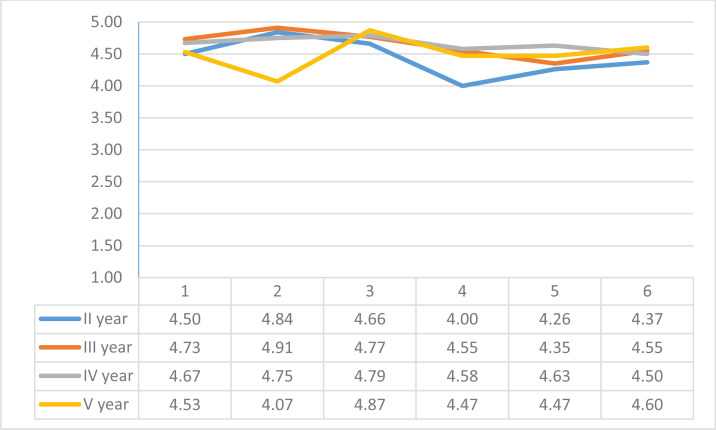
Student evaluation of supervision, based on six statements, Estonian Midwifery students (2016–2019)

Students answered questions related to the supervisor in the five-point system. The satisfaction of the third- and fourth-year students with the supervision was fairly stable. Second- and fifth-year students expressed more dissatisfaction. The students responded to the first statement: ‘The mentor devoted the necessary amount of time to me’, with an average rating of 4.60. Notably, 74.8% of respondents fully agreed with the validity of the statement, who rated it on a scale of point 5. This statement was rated the highest by third-year students (4.73) (Figure 1).

The rating for statement 2: ‘The supervisor's explanations were helpful to me’, was also highest in the case of the third-year students (4.91), while the fifth-year students rated this statement significantly lower (4.07). Statement 3: ‘The tutor answered my professional questions professionally’ was rated the highest by fifth-year students (4.87). The fifth-year students did not feel that the supervisors explained exactly what they felt was most important, while when the students asked questions themselves, they felt that the knowledge gained was very important.

The students rated statement 4: ‘The supervisor's expectations of me were in line with my skill level’, and 52% of students fully agreed with this statement. The analysis of the data showed that the agreement with the respective statement was the lowest among the second-year students (4.0). The answers given by the 3rd, 4th and 5th year students show that the further along with the studies, the higher the validity of the statement.

*‘They often don't know which year's students come to the internship, and then they don't know their skills either’*.

The biggest concern for second-year students was that when they go on an internship for the first time in the second academic year, the supervisors seem to expect certain skills and experiences that they do not yet have.

The students rated statement 5: ‘Did the supervisor give me the necessary feedback on the performance of the assignments’, which resulted in an average score of 4.43. The second-year students gave the lowest average rating.

*‘Both negative and positive performances were discussed with the supervisor.’*

Most fifth-year students (4.6) agreed to statement 6: ‘I did not get the impression that I was a burden to the supervisor’, while the second-year students felt the most that they could be a burden to the supervisor (4.37) (Figure 1).

### Satisfaction and evaluation of the work of the department and co-workers

**Figure 2:**
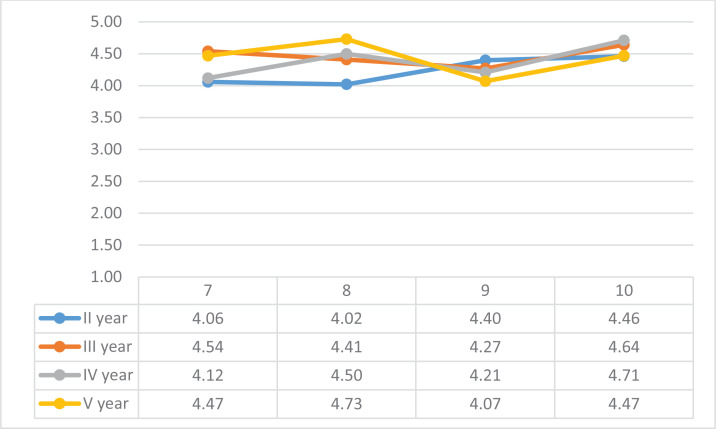
Evaluation of the work of the department and staff, Estonia 2016–2019

The students rated statement 7: ‘The department had a pleasant communication atmosphere’, with an average score of 4.27. The validity of the statement was assessed on a scale of point 5, with full agreement by 52%. The lowest average rating of the statement was by the second-year students (4.06), while third-year students agreed with the validity of the statement the most (average rating 4.54) (Figure 2). The comments revealed that the communication atmosphere is also influenced by the relations between the employees. One student also pointed out that the communication atmosphere could vary from day to day.

The students of the midwifery specialty of college evaluated statement 8: ‘The expectations of the co-workers for me corresponded to my level of skills’, the average rating of which was 4.33 on the basis of the collected questionnaires. By courses, the validity of the statement was assessed lower among second-year students. Almost a quarter (24%) of the second-year respondents rated the validity of the statement with a scale point of 2 or 3.

*‘Did not take into account the level of 2nd year and little experience at the beginning of the internship.’*

The students of the senior years found that the expectations of the co-workers were lower than expected.

*‘At first, they didn't trust me, but when I proved myself, I was allowed to operate independently.’*

The students evaluated statement 9: ‘I was taken advantage of, and I was used as an unpaid workforce for tasks not related to studies’. The average rating of the validity of the statement was 4.32. By study years, the attitude related to the validity of the statement was the lowest among fifth-year students. In the comparison of the study years, it is noticeable that the further into the studies, the students’ average assessment of the validity of the statement decreased.

*‘Even if I was taken advantage of it was with my kind permission. I'm in the hospital for learning and ready to do (almost) anything.’*

The average rating of statement 10: ‘I was sufficiently involved in the activities of the department’ was 4.58. By courses, the lowest average rating was given by second-year students.

*‘We were given a lot of opportunities to act. At times, I even got the impression that all the work was given to the interns. Which in itself was a good opportunity for manual training.’*

### Student satisfaction and evaluation of the internship

**Figure 3:**
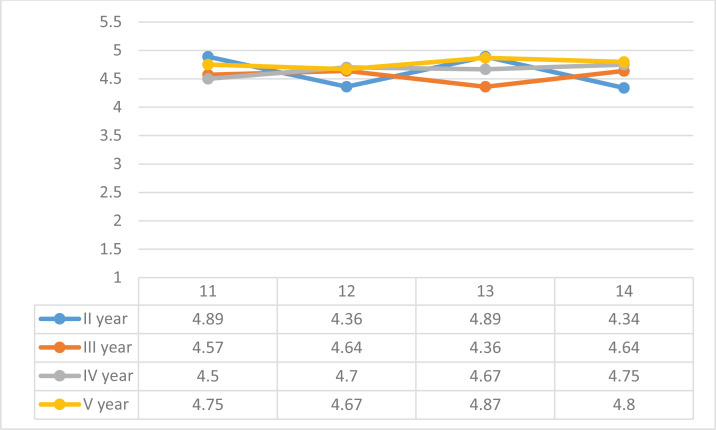
Student satisfaction and evaluation of the internship, Estonia 2016-2019

Students were asked to comment on statement 11: ‘I had set personal goals for myself when I went on internship’. Among the students, the validity of the statement was assessed by 4.65 points; 78.9% of students rated the statement on the scale by 5 points. Second-year students had the highest self-esteem (4.89). The fourth-year students had the lowest average rating (4.50) (Figure 3).

Next was also evaluated statement 12 that measured the achievement of the goals: ‘I achieved the goals of my internship to the extent suitable for me. Most of the students achieved their goals fully; 64.6% fully agreed with the statement. The lowest average rating of the statement was in the second year (4.36).

For statement 13: ‘I felt I was welcome to the internship’, the students’ average rating was 4.65; 76.7% of students fully agreed with the statement. The statement was rated the highest by the second-year students. The students answered statement 14: ‘I had enough opportunities for independent action’, with an average rating of 4.60; 73.2% of students fully agreed with the statement. Students’ assessment of the validity of the statement is on an increasing trend by study years. The highest average rating of the statement was in the 5th year (4.80). The lowest average rating of the statement was in the second year (4.34).

## DISCUSSION

Mentors are expected to have certain personal qualities such as friendliness, a sense of humor, patience, consistency, rationality, and openness^16,17^. In addition to personal qualities, the ability to give feedback, work experience, a positive attitude towards life, and giving time to the intern are important in supervision^18^. This study shows that mentors, in turn, expect students to be active, prepared, punctual, interested in self-improvement, and willing to apply previously acquired theoretical knowledge in the internship base. At the same time, it has been found that a motivated mentor, in turn, motivates the student to study^19^. It was also found that a mentor can make students more active by involving and motivating them^20^. It can be said here that motivation is reciprocal.

This study shows that students often feel that they are a burden, perceive little feedback from the internship supervisor and that the internship supervisor has too high/low expectations for them. The results show that as students progress and their skills and self-confidence increase, interns appreciate that mentors' expectations are better matched to their skills. Licqurish and Seibold^21^ have stated that long-term cooperation with one internship supervisor allows both parties to understand each other’s realistic expectations more realistically, gives the mentor a better overview of the intern's skills and the opportunity to provide more in-depth feedback.

Being a mentor requires certain skills and personal qualities, and therefore there is a constant debate about whether all midwives should be mentors or whether mentoring should belong to a smaller selected group^12,22^. This study also highlights the problem of students being mentored by mentors who do not really want to do so. The International Code of Ethics for Midwives states that the transfer of professional knowledge to future generations is essential for the advancement of the field^7^. The role of a mentor is demanding, complex, and can be frustrating, especially if the mentor performs mentoring reluctantly. The mentor is often expected to have the necessary skills without relevant training. Most health professionals have acquired professional knowledge and skills, but knowledge of teaching and supervision is lacking. Mentoring plays an important role in the organization of activities. Therefore, higher education institutions and employers must ensure that their staff have the opportunity to participate in training on mentoring issues^17^.

Due to lack of time, it is difficult for mentors to fulfill their job responsibilities and be a mentor to the intern at the same time. Shakespeare and Webb^23^ pointed out that the work of mentors in a hospital needs to be adapted in such a way that it is possible to commit to mentoring responsibilities. Due to lack of time, a fixed time for the intern could be a part of the work organization in Estonia as well. This mentor–intern time is set out in the standards set by the Standards to Support Learning and Assessment in Practice 2015 in the UK. Midwives are provided with one hour per week per intern, which ensures that the mentor has the opportunity to provide continuous feedback to the intern. Mentors think that a fixed time must be provided by the hospital and must be part of working hours. It is also found that bonuses motivate and compensate a little for a significant increase in workload caused by the supervision of an intern.

Due to the internationalization of higher education, one of the challenges of supervision is the number of international students. Under the guidance of international interns, establishing a collaborative relationship is perceived as a challenge due to cultural and/or linguistic differences. Feedback from international students has shown that it is difficult for mentors to build a working relationship, and therefore they have a negative attitude. There are no guidelines for mentors that would positively support the mentoring of international students. Mentors find instruction and teaching in a foreign language exhausting and stressful, and it is also difficult to fill in documentation^24^. Similarly, this study found that there are positive aspects to supervising international students, such as thorough feedback from students and a varied and developing experience for the supervisor. But also, negative aspects such as language barriers, cultural specificities, and uneven levels. The increase in the number of international students in the field of healthcare inevitably brings problems, and there is reason to believe that the problems associated with international students will worsen if they are ignored.

### Strengths and limitations

The study has been conducted in an Estonian women’s clinic, where about 30% of all births in Estonia take place. This clinic has the largest number of births per year in the country. The strength of the study is that students were interviewed for three years, and all students who had completed an internship at a given hospital were included (student years 1–5). The midwives’ focus group interviews provided positive feedback on the mentors’ thoughts and suggestions on this topic. The disadvantage of the present study is that the study was conducted in the context of one school midwifery curriculum student and one hospital. At the same time, it must be taken into account that Estonia is a small country, and midwives are taught in only two higher education institutions in the country, and 13 hospitals have a maternity ward. However, this hospital has the largest number of births in the country. In the future, the involvement of other higher education institutions and other hospitals in this topic could also be considered.

## CONCLUSIONS

Mentors are aware that they have a responsibility to instruct, train and evaluate interns, and they believe that a supportive environment is essential for a successful internship. The internal climate of the department is influenced by the hospital staff, patients, and trainees. The greatest difficulty for the mentors is posed by lack of time and therefore increased workload. The satisfaction of midwifery students with the internship depends on the environment and internal climate of the internship base. Student satisfaction with the mentor and co-workers determines how successful the students perceive the collaboration to be. It is sometimes a problem for students, especially junior students, that they are expected to have a higher level of skills than their acquired knowledge allows. The study sends an important message to the midwifery mentors to enhance the quality and motivation of internships, and reciprocal learning and teaching are important for both mentors and midwifery students.

## Data Availability

The data supporting this research are available from the authors on reasonable request.
